# Supplementary material for: Modeling glioblastoma heterogeneity as a dynamic network of cell states
Source: Mol Syst Biol. 2021 Sep 16;17(9):e10105. doi: 10.15252/msb.202010105 (PMC8444284; doi:10.15252/msb.202010105)
Supplement: Supplementary file 6 — Source Data for Figure 5 [file MSB-17-e10105-s004.zip › Figure5A_sourcedata/GSEA_3017/nosigdb_stateF.GseaPreranked.1622795015644/GAROFANO_IAVARONE_MTC_SUBTYPE.html]

Details for gene set GAROFANO\_IAVARONE\_MTC\_SUBTYPE[GSEA]

|  || Dataset | state33017 |
| Phenotype | NoPhenotypeAvailable |
| Upregulated in class | na\_pos |
| GeneSet | GAROFANO\_IAVARONE\_MTC\_SUBTYPE |
| Enrichment Score (ES) | 0.50183487 |
| Normalized Enrichment Score (NES) | 2.9666212 |
| Nominal p-value | 0.0 |
| FDR q-value | 0.0 |
| FWER p-Value | 0.0 |
Table: GSEA Results Summary

  

Fig 1: Enrichment plot: GAROFANO\_IAVARONE\_MTC\_SUBTYPE      
 Profile of the Running ES Score & Positions of GeneSet Members on the Rank Ordered List

  

| PROBE | GENE SYMBOL | GENE\_TITLE | RANK IN GENE LIST | RANK METRIC SCORE | RUNNING ES | CORE ENRICHMENT || 1 | CDKN1A |  |  | 4 | 2.089 | 0.0474 | Yes |
| 2 | CLU |  |  | 5 | 1.852 | 0.0911 | Yes |
| 3 | HEPACAM |  |  | 13 | 1.356 | 0.1198 | Yes |
| 4 | RPS27L |  |  | 33 | 1.025 | 0.1353 | Yes |
| 5 | PLTP |  |  | 35 | 1.020 | 0.1588 | Yes |
| 6 | SLC4A4 |  |  | 40 | 0.980 | 0.1801 | Yes |
| 7 | ALDH3A2 |  |  | 43 | 0.958 | 0.2018 | Yes |
| 8 | CMBL |  |  | 49 | 0.935 | 0.2215 | Yes |
| 9 | MLC1 |  |  | 58 | 0.896 | 0.2390 | Yes |
| 10 | ASCC3 |  |  | 60 | 0.873 | 0.2591 | Yes |
| 11 | TSC22D4 |  |  | 64 | 0.855 | 0.2779 | Yes |
| 12 | NDFIP1 |  |  | 74 | 0.814 | 0.2929 | Yes |
| 13 | AQP4 |  |  | 84 | 0.789 | 0.3074 | Yes |
| 14 | PHPT1 |  |  | 109 | 0.738 | 0.3138 | Yes |
| 15 | FDXR |  |  | 120 | 0.716 | 0.3260 | Yes |
| 16 | BTG2 |  |  | 124 | 0.708 | 0.3413 | Yes |
| 17 | RHOU |  |  | 145 | 0.679 | 0.3482 | Yes |
| 18 | TMEM59L |  |  | 153 | 0.661 | 0.3605 | Yes |
| 19 | DDR1 |  |  | 159 | 0.649 | 0.3735 | Yes |
| 20 | DTNA |  |  | 176 | 0.617 | 0.3807 | Yes |
| 21 | FBXO22 |  |  | 205 | 0.585 | 0.3816 | Yes |
| 22 | TRIAP1 |  |  | 208 | 0.584 | 0.3945 | Yes |
| 23 | HIGD1A |  |  | 212 | 0.580 | 0.4068 | Yes |
| 24 | SCHIP1 |  |  | 233 | 0.566 | 0.4109 | Yes |
| 25 | BBOX1 |  |  | 241 | 0.559 | 0.4209 | Yes |
| 26 | CA2 |  |  | 243 | 0.558 | 0.4336 | Yes |
| 27 | TMEM131 |  |  | 246 | 0.556 | 0.4458 | Yes |
| 28 | NLRP1 |  |  | 257 | 0.547 | 0.4541 | Yes |
| 29 | LFNG |  |  | 275 | 0.530 | 0.4587 | Yes |
| 30 | MTTP |  |  | 285 | 0.523 | 0.4669 | Yes |
| 31 | PPP2R2B |  |  | 311 | 0.505 | 0.4673 | Yes |
| 32 | ACYP2 |  |  | 317 | 0.502 | 0.4769 | Yes |
| 33 | C18orf32 |  |  | 335 | 0.495 | 0.4807 | Yes |
| 34 | HIGD2A |  |  | 414 | 0.464 | 0.4558 | Yes |
| 35 | PRCP |  |  | 426 | 0.460 | 0.4616 | Yes |
| 36 | TMEM205 |  |  | 452 | 0.449 | 0.4606 | Yes |
| 37 | METTL8 |  |  | 458 | 0.448 | 0.4689 | Yes |
| 38 | JUND |  |  | 478 | 0.442 | 0.4706 | Yes |
| 39 | RAB9A |  |  | 489 | 0.438 | 0.4763 | Yes |
| 40 | LMO2 |  |  | 497 | 0.436 | 0.4833 | Yes |
| 41 | ZNF561 |  |  | 511 | 0.430 | 0.4875 | Yes |
| 42 | PFKM |  |  | 547 | 0.416 | 0.4812 | Yes |
| 43 | KCNN2 |  |  | 556 | 0.412 | 0.4872 | Yes |
| 44 | PTPMT1 |  |  | 589 | 0.401 | 0.4820 | Yes |
| 45 | IRS2 |  |  | 595 | 0.400 | 0.4891 | Yes |
| 46 | IER5 |  |  | 605 | 0.396 | 0.4943 | Yes |
| 47 | SEMA6D |  |  | 637 | 0.388 | 0.4892 | Yes |
| 48 | RGS6 |  |  | 645 | 0.386 | 0.4951 | Yes |
| 49 | AIG1 |  |  | 651 | 0.385 | 0.5018 | Yes |
| 50 | SYT17 |  |  | 699 | 0.371 | 0.4890 | No |
| 51 | CCDC102B |  |  | 702 | 0.370 | 0.4968 | No |
| 52 | WNT7A |  |  | 735 | 0.362 | 0.4906 | No |
| 53 | MASP1 |  |  | 755 | 0.357 | 0.4903 | No |
| 54 | PTPRA |  |  | 777 | 0.352 | 0.4889 | No |
| 55 | RORA |  |  | 783 | 0.351 | 0.4949 | No |
| 56 | CDS2 |  |  | 879 | 0.330 | 0.4590 | No |
| 57 | NIPAL3 |  |  | 906 | 0.324 | 0.4547 | No |
| 58 | USP2 |  |  | 953 | 0.317 | 0.4410 | No |
| 59 | ROBO2 |  |  | 963 | 0.316 | 0.4443 | No |
| 60 | PJA2 |  |  | 986 | 0.312 | 0.4415 | No |
| 61 | ALDH6A1 |  |  | 987 | 0.311 | 0.4489 | No |
| 62 | EXD2 |  |  | 1041 | 0.302 | 0.4316 | No |
| 63 | INPP5D |  |  | 1069 | 0.297 | 0.4262 | No |
| 64 | TSPAN3 |  |  | 1071 | 0.297 | 0.4327 | No |
| 65 | TMEM68 |  |  | 1075 | 0.296 | 0.4383 | No |
| 66 | ZNF181 |  |  | 1133 | 0.287 | 0.4189 | No |
| 67 | FBXL17 |  |  | 1136 | 0.287 | 0.4247 | No |
| 68 | UQCRB |  |  | 1191 | 0.280 | 0.4065 | No |
| 69 | CKB |  |  | 1200 | 0.279 | 0.4094 | No |
| 70 | FZD3 |  |  | 1214 | 0.277 | 0.4099 | No |
| 71 | TRIM4 |  |  | 1281 | 0.271 | 0.3860 | No |
| 72 | KIF21A |  |  | 1298 | 0.269 | 0.3849 | No |
| 73 | TMED1 |  |  | 1309 | 0.267 | 0.3866 | No |
| 74 | GRIK1 |  |  | 1329 | 0.265 | 0.3841 | No |
| 75 | MAPK8IP1 |  |  | 1340 | 0.263 | 0.3857 | No |
| 76 | SPATA7 |  |  | 1390 | 0.258 | 0.3693 | No |
| 77 | C1orf53 |  |  | 1436 | 0.254 | 0.3545 | No |
| 78 | ZNF626 |  |  | 1439 | 0.254 | 0.3596 | No |
| 79 | OSBPL6 |  |  | 1451 | 0.252 | 0.3605 | No |
| 80 | ZYX |  |  | 1745 | -0.338 | 0.2337 | No |
Table: GSEA details [plain text format]

  

Fig 2: GAROFANO\_IAVARONE\_MTC\_SUBTYPE: Random ES distribution      
 Gene set null distribution of ES for **GAROFANO\_IAVARONE\_MTC\_SUBTYPE**
